# Simple biomarkers based on CRP and albumin predict clinical outcomes in adult patients with T-cell acute lymphoblastic leukaemia

**DOI:** 10.3389/fnut.2025.1708810

**Published:** 2026-01-08

**Authors:** Aiwen Li, Jun Wen, Xianfang Shao, Qiuju Liu

**Affiliations:** 1 Department of Haematology, Cancer Center, The First Hospital of Jilin University, Changchun, China; 2 Department of Intensive Care Medicine, Zibo Central Hospital, Zibo, China

**Keywords:** acute lymphocytic leukaemia, albumin, C-reactive protein, fibrinogen, prognosis

## Abstract

**Introduction:**

Inflammation and malnutrition adversely impact outcomes in patients with various malignancies. Composite indices such as the C-reactive protein/albumin ratio (CAR), the CRP × fibrinogen/albumin ratio (CFA), and the modified Glasgow Prognostic Score (mGPS) integrate these parameters, although their prognostic role in T-cell acute lymphoblastic leukaemia (T-ALL) remains underexplored.

**Methods:**

In this single-centre retrospective study, 74 adults with T-ALL were included. CAR, CFA, and mGPS were calculated at diagnosis. Receiver operating characteristic curve analysis revealed the optimal cut-off values for the CAR (0.387) and CFA (0.396). Patients were stratified into low- and high-risk groups. Endpoints included rates of complete remission/complete remission with incomplete haematologic recovery (CR/CRi) at end-of-induction (EOI), minimal residual disease (MRD), overall survival (OS), and progression-free survival (PFS).

**Results:**

Patients with low CAR, low CFA, or mGPS0 achieved significantly higher rates of CR/CRi (all *p* < 0.05) and MRD < 0.1% (all *p* < 0.05) at EOI. These low-risk groups also exhibited significantly fewer chemotherapy cycles to achieve the first CR/CRi (all *p* < 0.001) and shorter time to achieve MRD negativity (all *p* < 0.001). Survival analysis revealed significantly longer OS and PFS in the low-risk group (all *p* < 0.05). Multivariate analysis revealed high CAR (*p* = 0.004) and MRD positivity ≥0.1% at EOI (*p* = 0.043) as independent predictors of poor OS. Subgroup analysis indicated that allogeneic hematopoietic stem cell transplantation significantly improved survival only in high-risk patients.

**Conclusion:**

Pretreatment CAR, CFA, and mGPS are robust, accessible prognostic biomarkers in adults with T-ALL. Their integration into initial risk assessment could help guide personalized treatment strategies, including the identification of high-risk patients who may derive greater benefit from aggressive interventions.

## 1 Introduction

T-cell acute lymphoblastic leukaemia (T-ALL) is an aggressive haematologic malignancy derived from early T-cell progenitors. Surveillance, Epidemiology, and End Results (SEER) Program registry data show an overall incidence of T-ALL of approximately 1.6 per 100,000 population (1–3). While 80% of ALL occurs in children, it is a rare but devastating disorder in adults, with an unfavorable 5-year overall survival rate of 41% (4, 5). Because of this rarity, it is difficult to conduct large-scale clinical studies. Consequently, the prognostic stratification of adult patients with T-ALL remains a hot topic, and progress in its treatment is limited. Current risk stratification in T-ALL relies primarily on clinical metrics such age, immunophenotype, central nervous system leukaemia (CNSL), and minimal residual disease (MRD), and genomic landscape is a trend with a poor accessibility (6, 7). However, these factors cannot independently predict outcomes beyond response to therapy in multivariate risk models, highlighting the need for additional biomarkers to refine risk assessment and guide treatment strategies.

The inflammatory response and nutritional status are key drivers of malignancy progression (8, 9). Inflammatory-nutritional markers including the modified Glasgow Prognostic Score (mGPS), the CRP × fibrinogen/albumin ratio (CFA) and the C-reactive protein-albumin ratio (CAR) are closely associated with prognosis in patients with various solid tumors and some hematological malignancies (10–15). Nevertheless, their predictive significance in T-ALL remains unexplored.

Therefore, this study aimed to evaluate the prognostic utility of the CAR, CFA, and mGPS in relation to induction response, MRD dynamics, and survival in a well-characterized cohort of adult T-ALL patients. To our knowledge, this represents the first comprehensive analysis of these composite biomarkers specifically in T-ALL.

## 2 Methods

### 2.1 Patients

This single-centre retrospective cohort study included patients aged ≥14 years who were diagnosed with T-ALL and who underwent treatment at our centre between January 2013 and June 2024. Among 108 initially identified T-ALL patients, 34 were excluded on the basis of the following criteria: diagnosis at external institutions (7 patients), absence of systemic treatment (9 patients), or lack of baseline serum albumin and/or C-reactive protein (CRP) measurements prior to induction chemotherapy (18 patients). Consequently, 74 patients were included in the final study cohort. This study was conducted in compliance with ethical principles based on the Declaration of Helsinki and was approved by the Institutional Review Board of the first hospital of Jilin University (No. 2024-1324). Written consent was obtained from each patient for participation in the study.

### 2.2 Treatment

All patients underwent induction chemotherapy consisting of vincristine or vindesine, anthracyclines, and glucocorticoids. Patients who achieved complete remission (CR) underwent consolidation therapy with either hematopoietic stem cell transplantation (HSCT) or chemotherapy followed by sequential maintenance therapy on the basis of clinical situation. Patients who did not achieve CR received reinduction chemotherapy. The minimum follow-up period was 12 months or until death for all participants.

### 2.3 Definitions

Treatment response was assessed according to the European LeukemiaNet (ELN) working group criteria (16). Complete remission (CR) was defined as less than 5% bone marrow blasts, no extramedullary disease, an absolute neutrophil count >1.0 × 10^9^/L, a platelet count >100 × 10^9^/L, and a lack of red cell transfusions. CR with incomplete haematologic recovery (CRi) was defined as meeting all of the CR criteria except for residual neutropenia <1.0 × 10^9^/L or thrombocytopenia <100 × 10^9^/L. Progressive disease (PD) is defined as the emergence of blasts in peripheral blood, a > 25% increase in absolute blast count (in peripheral blood or bone marrow), or the development of new extramedullary disease. Minimal residual disease (MRD) negativity was defined as MRD undetectable by flow cytometry at a sensitivity level of 0.01%.

In addition to gender and age, we collected information on each patient at diagnosis of ALL; blood sample tests before treatment (CRP (mg/L), albumin (g/L), fibrinogen (g/L)), the presence of fever over 38 °C, the usage of anti-infective therapy. The C-reactive protein–albumin ratio (CAR) was calculated as the serum CRP concentration (mg/L) divided by the serum albumin concentration (g/L) (13). The CFA was calculated as serum CRP (mg/L) multiplied by fibrinogen (g/L), divided by serum albumin (g/L) (11). The modified Glasgow Prognostic Score (mGPS) was calculated by assigning one point for elevated CRP (≥10 mg/L) and in patients with elevated CRP a second point for reduced albumin (<35 g/L) (12). Overall survival (OS) was defined as the time from treatment initiation to death from any cause or last follow-up contact. Progression-free survival (PFS) was defined as the time from treatment initiation to documented PD or death from any cause.

### 2.4 Statistical analysis

Descriptive statistics were used to summarize baseline patient and disease characteristics. To determine the optimal prognostic thresholds, receiver operating characteristic (ROC) curve analysis was employed to determine the ability of the CAR and the CFA ratio to predict one-year mortality in T-ALL patients. Survival outcomes, including OS and PFS, were visualized using Kaplan–Meier survival curves. Univariate and multivariate Cox proportional hazards regression analyses were conducted to identify factors significantly associated with prognosis in T-ALL patients. A two-tailed test was used for all analyses, with a statistical significance level set at *p* < 0.05.

## 3 Results

### 3.1 Patient characteristics

**Figure 1:**
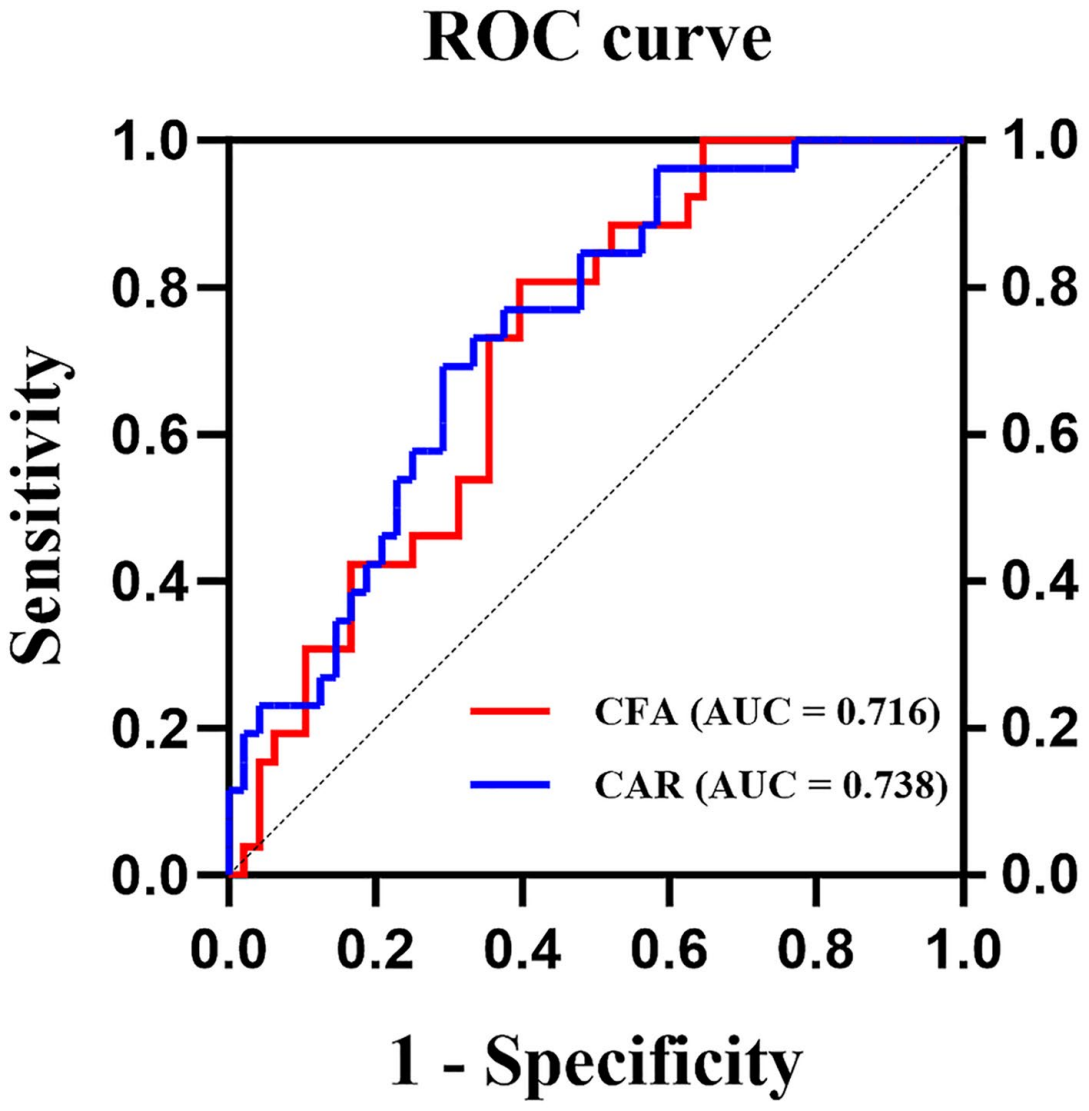
Analysis of ROC curves of CFA and CAR for predicting one-year mortality. The ROC analysis demonstrated that CFA achieved an AUC of 0.716 (optimal cut-off: 0.396; sensitivity: 80.8%; specificity: 60.4%), while CAR yielded a higher AUC of 0.738 (optimal cut-off: 0.387; sensitivity: 69.2%; specificity: 70.8%).

The baseline characteristics and laboratory parameters of the 74 enrolled patients are presented in Table 1. The median CRP, median albumin, and median fibrinogen concentrations were 7.10 mg/L (range: 0.24–102 mg/L), 36.5 g/L (range: 25.0–45.5 g/L), and 2.58 g/L (range: 0.60–6.79 g/L), respectively. The CAR and CFA were subsequently calculated. To evaluate their prognostic utility for survival, receiver operating characteristic (ROC) curves were constructed, identifying optimal cut-off values of 0.396 for the CFA (AUC: 0.716; sensitivity: 80.8%; specificity: 60.4%) and 0.387 for the CAR (AUC: 0.738; sensitivity: 69.2%; specificity: 70.8%) (Figure 1). Patients were then stratified into groups on the basis of these cut-off values. Differences in clinical characteristics and treatment outcomes between the stratified groups were compared, as detailed in Table 1. The patient groups (high vs. low CAR, high vs. low CFA, and mGPS 0, 1, vs. 2) were well-balanced at baseline, with no statistically significant differences (*p* > 0.05) in terms of age, gender, BMI at diagnosis, immunophenotype, genetic mutations, pre-induction fever, antibiotic use, or receipt of hematopoietic stem cell transplantation (Table 1).

**Table 1:** Patient and disease characteristics.

Characteristics	Total	CAR		*p*-value	CFA		*p*-value	mGPS			*p*-value
		Low-CAR (<0.387)	High-CAR (≥0.387)		Low-CFA (<0.396)	High-CFA (≥0.396)		mGPS0	mGPS1	mGPS2	
Number of patients, *n* (%)	74	42 (56.8%)	32 (43.2%)	–	34 (45.9%)	40 (54.1%)	–	38 (51.4%)	15 (20.3%)	21 (28.4%)	–
Age (years), *n* (%)				0.090			0.230				0.275
<35	46 (62.2%)	30 (71.4%)	16 (50%)		24 (70.6%)	22 (55.0%)		27 (71.1%)	8 (53.3%)	11 (52.4%)	
≥35	28 (37.8%)	12 (28.6%)	16 (50%)		10 (29.4%)	18 (45.0%)		11 (28.9%)	7 (46.7%)	10 (47.6%)	
Gender, *n* (%)				0.194			0.190				0.118
Male	54 (73.0%)	28 (66.7%)	26 (81.3%)		22 (64.7%)	32 (80.0%)		25 (65.8%)	14 (93.3%)	15 (71.4%)	
Female	20 (27.0%)	14 (33.3%)	6 (18.8%)		12 (35.3%)	8 (20.0%)		13 (34.2%)	1 (6.7%)	6 (28.6%)	
WBC (10^9^/L), *n* (%)				0.005			0.004				0.017
WBC < 100	52 (70.3%)	24 (57.1%)	28 (87.5%)		18 (52.9%)	34 (85.0%)		21 (55.3%)	13 (86.7%)	18 (85.7%)	
WBC ≥ 100	22 (29.7%)	18 (42.9%)	4 (12.5%)		16 (47.1%)	6 (15.0%)		17 (44.7%)	2 (13.3%)	3 (14.3%)	
HGB (g/L), median (range)	94 (48–173)	111 (48–173)	80 (50–162)	0.070	99 (48–173)	89 (50–162)	0.262	111 (48–173)	96 (52–146)	81 (50–162)	0.259
PLT (10^9^/L), median (range)	62 (4–382)	54 (7–382)	67.5 (4–296)	0.462	70 (7–382)	61.5 (4–296)	0.925	60 (7–382)	64 (8–152)	61 (4–296)	0.905
CRP (mg/L), median (range)	7.10 (0.24–102)	3.23 (0.24–14.88)	33.09 (10.99–102)	<0.001	2.55 (0.24–7.06)	22.48 (4.58–102)	<0.001	3.02 (0.24–7.10)	18.90 (13.09–78.6)	33.68 (10.99–102)	<0.001
ALB (g/L), median (range)	36.5 (25.0–45.5)	37.5 (27.8–45.5)	32.8 (25.0–44.6)	0.004	37.5 (27.8–45.5)	33.85 (25.0–44.6)	0.006	37.5 (27.8–45.5)	39.0 (35.0–44.6)	31.2 (25.0–34.7)	<0.001
Fib (g/L), median (range)	2.58 (0.60–6.79)	2.11 (0.73–5.80)	2.90 (0.60–6.79)	0.025	2.02 (0.73–5.80)	2.94 (0.60–6.79)	<0.001	2.03 (0.73–5.80)	3.14 (0.60–6.32)	2.79 (1.64–6.79)	0.009
BMI (kg/m^2^), median (range)	23.2 (14.5–33.7)	23.0 (14.5–32.0)	23.4 (18.3–33.7)	0.464	22.3 (14.5–32.0)	23.4 (17.5–33.7)	0.259	23.0 (14.5–32.0)	23.5 (18.7–29.1)	23.3 (18.3–33.7)	0.573
Immunophenotype, *n* (%)				0.254			0.080				0.181
ETP-ALL	14 (18.9%)	6 (14.3%)	8 (25.0%)		4 (11.8%)	10 (25.0%)		5 (13.2%)	6 (40.0%)	3 (14.3%)	
Pro/Pre-T-ALL	32 (43.2%)	16 (38.1%)	16 (50%)		12 (35.3%)	20 (50.0%)		14 (36.8%)	5 (33.3%)	13 (61.9%)	
Cortical/Medullary T-ALL	14 (18.9%)	10 (23.8%)	4 (12.5%)		8 (23.5%)	6 (15.0%)		9 (23.7%)	2 (13.3%)	3 (14.3%)	
Unclassified	14 (18.9%)	10 (23.8%)	4 (12.5%)		10 (29.4%)	4 (10.0%)		10 (26.3%)	2 (13.3%)	2 (9.5%)	
Genetics^1^, (%)											
*NOTCH1*	30/61 (49.2%)	17/36 (47.2%)	13/25 (52.0%)	0.797	15/30 (50.0%)	15/31 (48.4%)	1.000	16/33 (48.5%)	7/13 (53.8%)	7/15 (46.7%)	1.000
*FBWX7*	8/61 (13.1%)	5/36 (13.9%)	3/25 (12.0%)	1.000	4/30 (13.3%)	4/31 (12.9%)	1.000	5/33 (15.2%)	1/13 (7.7%)	2/15 (13.3%)	0.886
*RAS*	14/61 (23.0%)	6/36 (16.7%)	8/25 (32.0%)	0.219	6/30 (20.0%)	8/31 (25.8%)	0.762	6/33 (18.2%)	4/13 (30.8%)	4/15 (26.7%)	0.606
*PTEN*	9/61 (14.8%)	7/36 (19.4%)	2/25 (8.0%)	0.286	6/30 (20%)	3/31 (9.7%)	0.301	7/33 (21.2%)	0/13 (0%)	2/15 (13.3%)	0.238
Temperature ≥38.5 °C pre-IC, *n* (%)	15 (20.3%)	6 (14.3%)	9 (28.1%)	0.120	5 (14.7%)	10 (25.0%)	0.211	5 (13.2%)	4 (26.7%)	6 (28.6%)	0.258
Anti-infective therapy pre-IC, *n* (%)	50 (67.6%)	26 (61.9%)	24 (75.0%)	0.317	21 (61.8%)	29 (72.5%)	0.455	23 (60.5%)	12 (80.0%)	15 (71.4%)	0.389
HSCT^2^, *n* (%)	24/63 (38.1%)	17/42 (40.5%)	7/21 (33.3%)	0.784	17/34 (50.0%)	7/29 (24.1%)	0.042	17/38 (44.7%)	4/12 (33.3%)	3/13 (23.1%)	0.365
CR/CRi attainment at EOI, *n* (%)	49 (66.2%)	36 (85.7%)	13 (40.6%)	<0.001	31 (91.2%)	18 (45.0%)	<0.001	34 (89.5%)	7 (46.7%)	8 (38.1%)	<0.001
MRD < 0.1% at EOI, *n* (%)	30 (40.5%)	24 (57.1%)	6 (18.8%)	<0.001	20 (58.8%)	10 (25.0%)	0.004	23 (60.5%)	4 (26.7%)	3 (14.3%)	<0.001
Relapse^3^, *n* (%)	26/63 (41.4%)	17/42 (40.5%)	9/21 (42.9%)	1.000	13/34 (38.3%)	13/29 (44.8%)	0.618	14/38 (36.8%)	5/12 (41.7%)	7/13 (53.8%)	0.592
Follow-up (months), median (range)	48.2 (0.58–130.4)	48.2 (7.1–130.4)	49.0 (0.58–127.7)	0.777	42.8 (7.1–120.1)	50.1 (0.58–130.4)	0.199	48.2 (7.1–130.4)	27.6 (0.58–74.9)	49.0 (1.1–127.7)	0.185

^1^Genetic mutation results were available for 61 of the 74 patients.

^2^Data on patients undergoing HSCT among the 63 achieving CR.

^3^Data from relapsed patients among 63 CR cases.

CAR, C-reactive protein to albumin ratio; CFA, CRP×fibrinogen/albumin ratio; mGPS, modified glasgow prognostic score; HGB, hemoglobin; PLT, platelet; CRP, C-reactive protein; ALB, albumin; Fib, fibrinogen; BMI, body mass index; ETP, early thymic precursor; IC, induction chemotherapy; HSCT, hematopoietic stem cell transplantation; CR, complete remission; CRi, CR with incomplete hematologic recovery; MRD, minimal residual disease; EOI, end-of-induction.

### 3.2 Early treatment response

#### 3.2.1 CR/CRi

**Figure 2:**
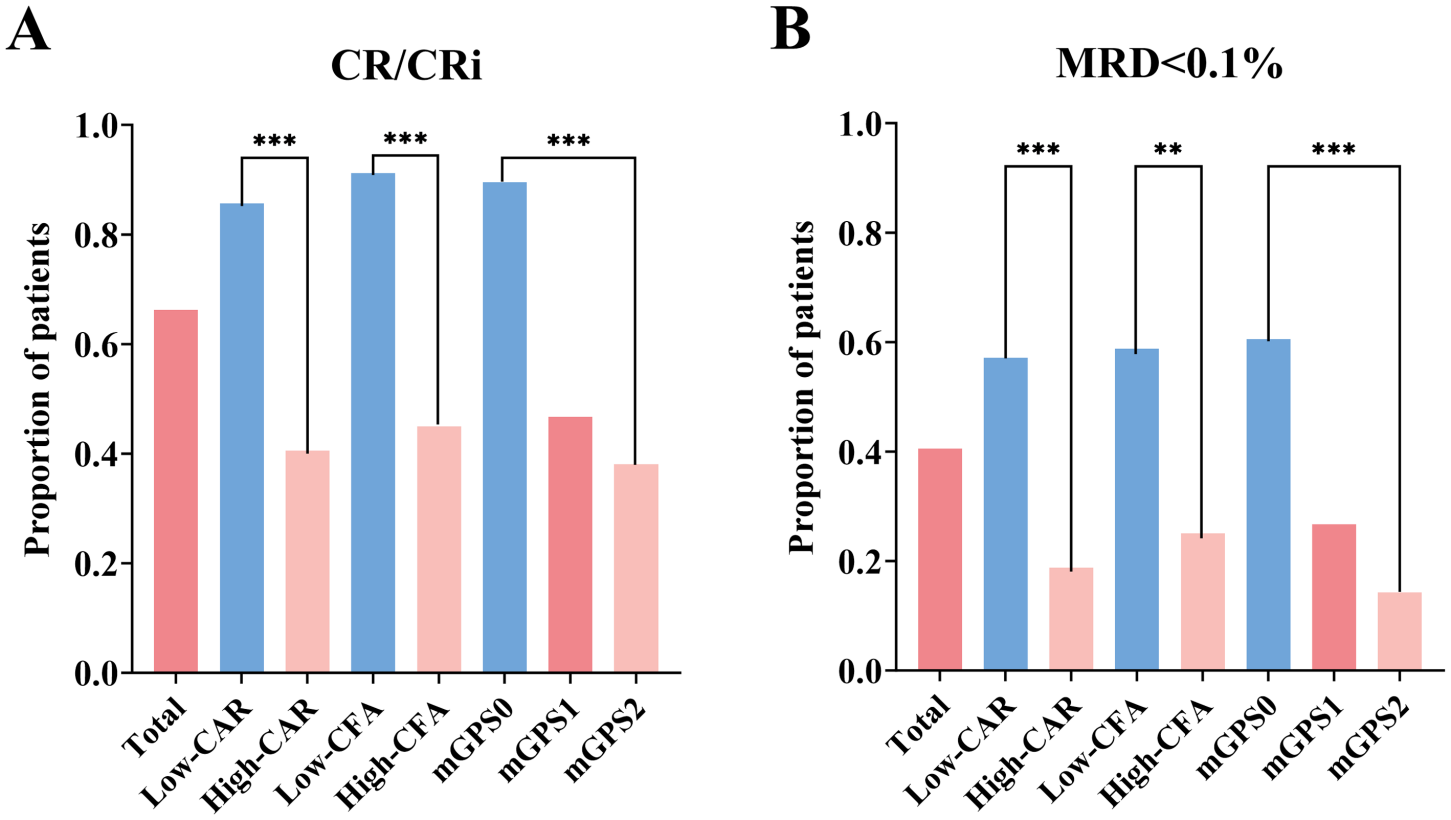
Rates of CR/CRi **(A)** and of MRD < 0.1% **(B)** at end of induction.

**Figure 3:**
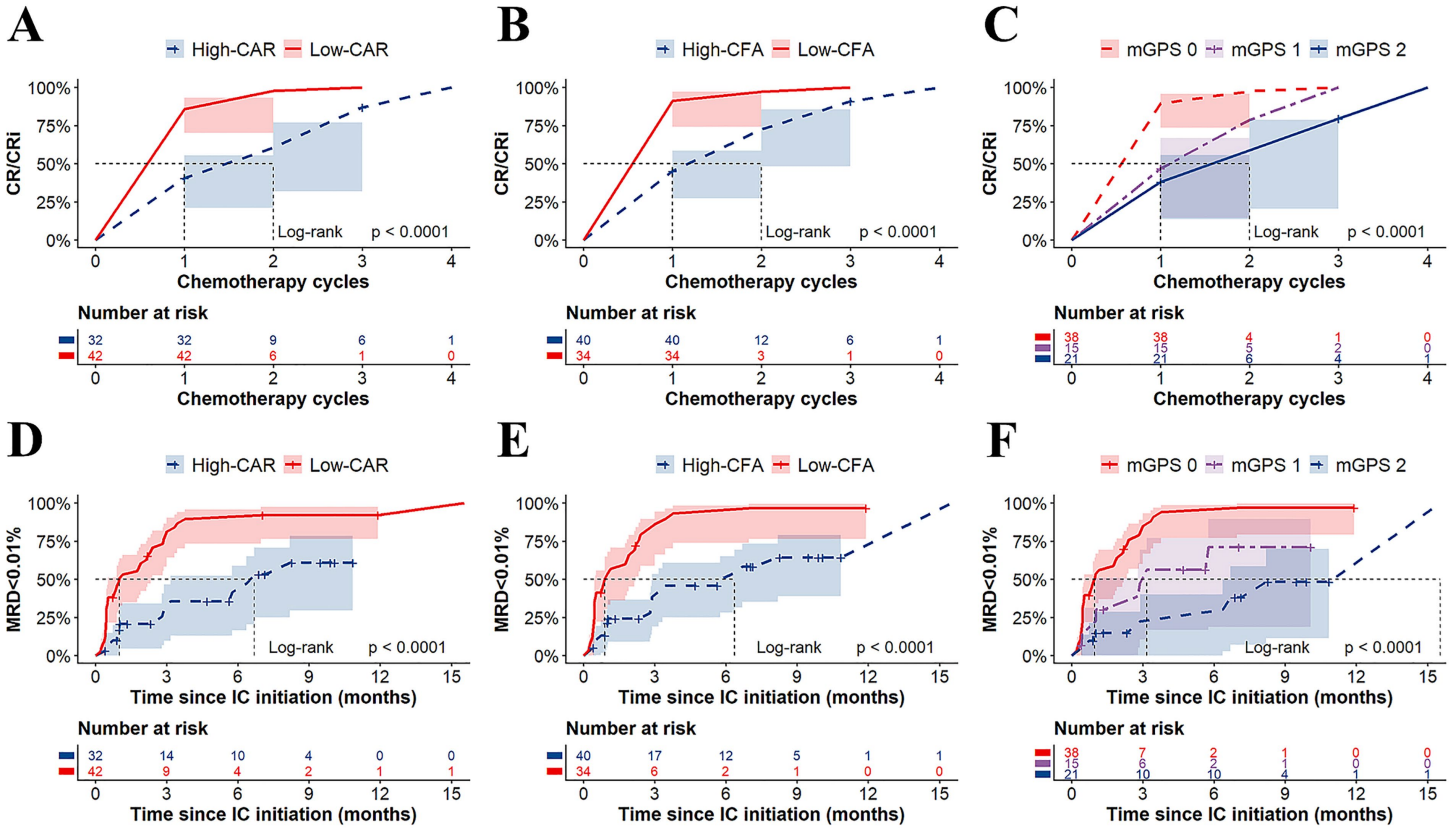
Time to CR/CRi and MRD negativity (<0.01%) by inflammatory indices. Kaplan–Meier curves showing the number of chemotherapy cycles required to achieve CR/CRi, stratified by the CAR **(A)**, CFA **(B)**, and mGPS **(C)** groups, respectively. Kaplan–Meier curves showing the time to MRD negativity (<0.01%), stratified by the CAR **(D)**, CFA **(E)**, and mGPS **(F)** groups, respectively.

At the end of induction (EOI), 49 of 74 patients (66.2%) achieved CR/CRi. When stratified by risk groups, the low-CAR group had significantly higher CR/CRi rates than the high-CAR group (85.7% vs. 40.6%; *p* < 0.001), and the low-CFA group had higher CR/CRi rates than the high-CFA group (91.2% vs. 45.0%; *p* < 0.001). Compared with the mGPS1 and mGPS2 groups, the mGPS0 group had significantly higher CR/CRi rates (89.5% vs. 46.7% vs. 38.1%; *p* < 0.001) (Figure 2A). Similarly, analysis of cycles to first CR/CRi in T-ALL patients revealed that the low-CAR, low-CFA, and mGPS0 groups required significantly fewer chemotherapy cycles than their high-risk counterparts did (all *p* < 0.001) (Figures 3A–C).

#### 3.2.2 MRD

At EOI, 30 of 74 patients (40.5%) achieved MRD < 0.1%. Stratification by risk group revealed significantly higher MRD < 0.1% rates in the low-CAR group than in the high-CAR group (57.1% vs. 18.8%; *p* < 0.001), in the low-CFA group than in the high-CFA group (58.8% vs. 25.0%; *p* = 0.004), and in the mGPS0 group than in both the mGPS1 and mGPS2 groups (60.5% vs. 26.7% vs. 14.3%; *p* < 0.001) (Figure 2B). Furthermore, the median time to first MRD negativity (<0.01%) was significantly shorter in the low-CAR group than in the high-CAR group (1.0 vs. 6.7 months; *p* < 0.001), in the low-CFA group than in the high-CFA group (0.9 vs. 6.4 months; *p* < 0.001), and in the mGPS0 group than in both the mGPS1 and mGPS2 groups (1.0 vs. 3.2 vs. 15.5 months; *p* < 0.001) (Figures 3D–F).

### 3.3 Survival outcomes

**Figure 4:**
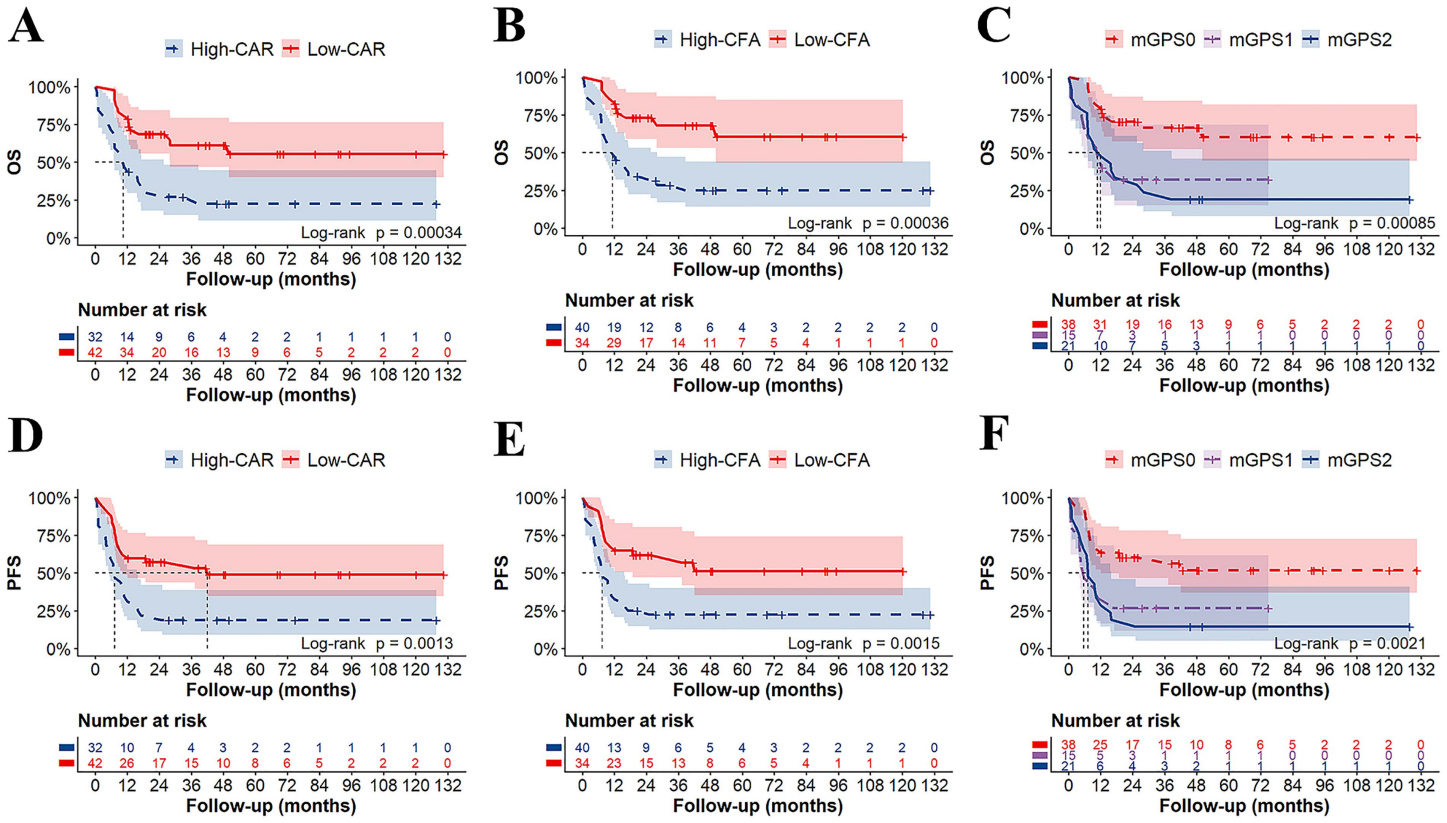
Kaplan–Meier Survival Analysis in 74 T-ALL Patients. The low-CAR group exhibited significantly longer median OS (not reached vs. 10.5 months; *p* < 0.001) **(A)** and PFS (42 vs. 7.2 months; *p* < 0.010) **(D)** than high-CAR group. The low-CFA group exhibited significantly longer median OS (not reached vs. 11.35 months; *p* < 0.001) **(B)** and PFS (not reached vs. 7.45 months; *p* < 0.010) **(E)** than high-CFA group. The mGPS0 group exhibited significantly longer median OS (not reached vs. 10.8 vs. 11.9 months; *p* < 0.001) **(C)** and PFS (not reached vs. 5.7 vs. 7.3 months; *p* < 0.010) **(F)** than mGPS1 and mGPS2.

**Figure 5:**
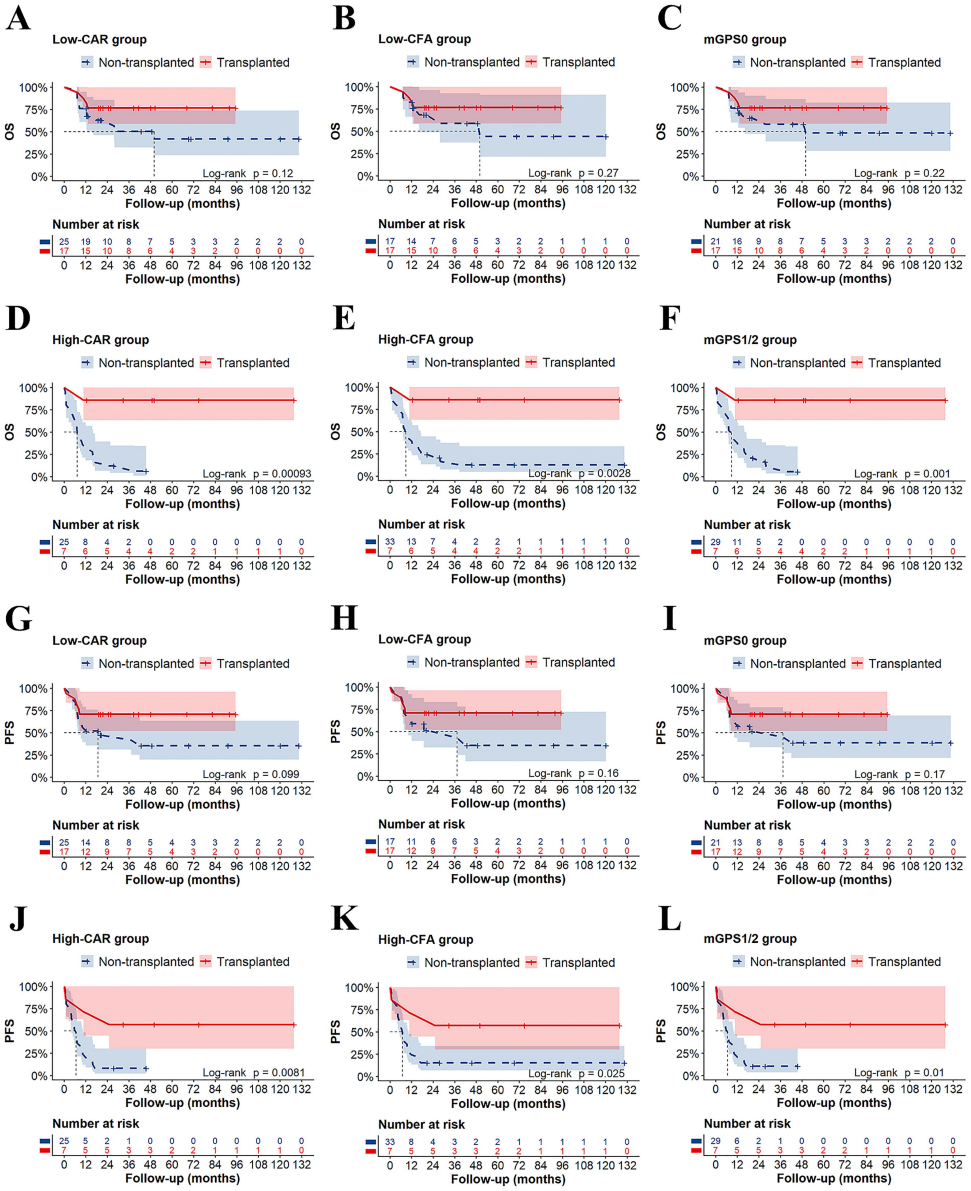
Subgroup analysis of OS and PFS in T-ALL patients. Low-CAR: HSCT did not confer a significant benefit in **(A)** OS (*p* > 0.05) or **(G)** PFS (*p* > 0.05). High-CAR: HSCT was associated with significantly improved **(D)** OS (*p* < 0.001) and **(J)** PFS (*p* < 0.01). Low-CFA: HSCT did not confer a significant benefit in **(B)** OS (*p* > 0.05) or **(H)** PFS (*p* > 0.05). High-CFA: HSCT was associated with significantly improved **(E)** OS (*p* < 0.01) and **(K)** PFS (*p* < 0.05). mGPS0: HSCT did not confer a significant benefit in **(C)** OS (*p* > 0.05) or **(I)** PFS (*p* > 0.05). mGPS1/2: HSCT was associated with significantly improved **(F)** OS (*p* < 0.01) and **(L)** PFS (*p* < 0.05).

**Figure 6:**
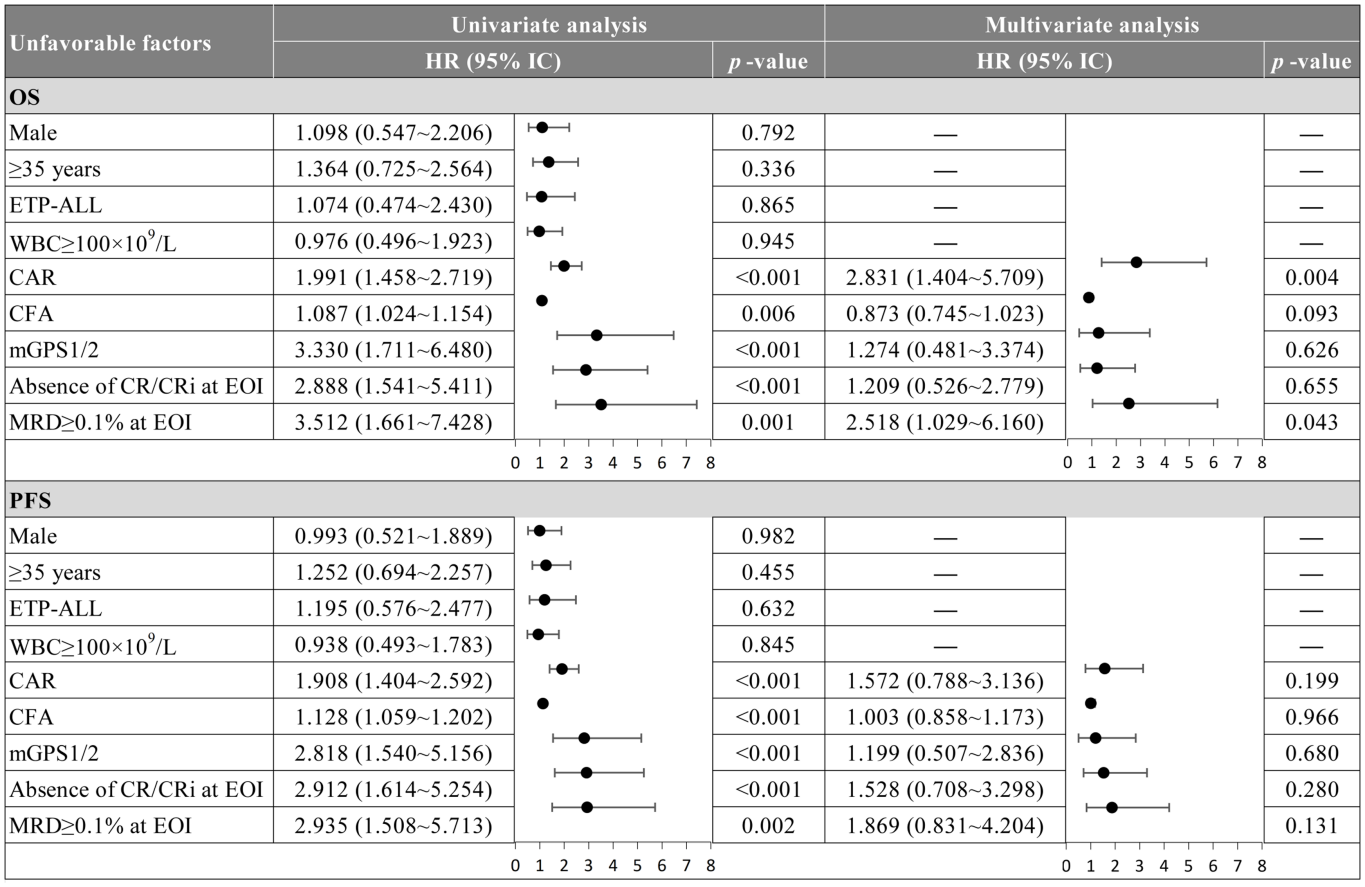
Multivariable Cox regression analysis of OS and PFS. OS, overall survival; PFS, progression-free survival; HR, hazard ratio; CAR, C-reactive protein to albumin ratio; CFA, CRP×fibrinogen/albumin ratio; mGPS, modified glasgow prognostic score; CR, complete remission; CRi, CR with incomplete hematologic recovery; ETP, early thymic precursor; MRD, minimal residual disease; EOI, end-of-induction.

Patients in the low-CAR group had significantly longer median OS (not reached vs. 10.5 months; *p* < 0.001) and median PFS (42 vs. 7.2 months; *p* < 0.010) than those in the high-CAR group (Figures 4A,D). Similarly, compared with patients in the high-CFA group, patients in the low-CFA group had significantly longer median OS (not reached vs. 11.35 months; *p* < 0.001) and median PFS (not reached vs. 7.45 months; *p* < 0.010) (Figures 4B,E). Furthermore, compared with patients with mGPS1 or mGPS2, patients with mGPS0 had significantly longer median OS (not reached vs. 10.8 vs. 11.9 months; *p* < 0.001) and median PFS (not reached vs. 5.7 vs. 7.3 months; *p* < 0.010), respectively (Figures 4C,F). Moreover, subgroup analysis revealed that in the low-risk group—comprising patients with low CAR, low CFA, and mGPS0—HSCT did not significantly improve OS or PFS (*p* > 0.05). In contrast, among the other high-risk subgroups, patients who underwent transplantation had significantly improved OS and PFS (*p* < 0.05) (Figures 5A–L). According to the univariate Cox analysis, a CAR, a CFA, an mGPS, the absence of a CR/CRi at the EOI, and an MRD ≥ 0.1% at the EOI were associated with both OS and PFS in T-ALL patients (all *p* < 0.05). To identify independent prognostic factors, we performed multivariable Cox proportional hazards regression analysis including variables that were significant in the univariate analysis. Multivariate Cox analysis revealed a CAR (HR = 2.831, 95% CI: 1.404–5.709, *p* = 0.004) and an MRD ≥ 0.1% at EOI (HR = 2.518, 95% CI: 1.029–6.160, *p* = 0.043) as independent risk factors for OS in T-ALL patients (Figure 6).

## 4 Discussion

This single-centre retrospective study demonstrated that CAR, CFA, and mGPS are strongly associated with treatment response and survival outcomes in adults with T-ALL. To our knowledge, this is the first study to comprehensively evaluate the prognostic utility of these composite inflammatory-nutritional indices specifically in an adult T-ALL cohort.

Chronic, dysregulated, and persistent inflammation is a well-established driver of tumor initiation, progression, and metastasis (17). Supporting this, Gower et al. recently identified a novel inflammatory T-lineage ALL subgroup and developed a prognostic gene signature, directly linking high inflammatory status to adverse outcomes in T-ALL patients (18). Concurrently, malnutrition—defined as a state of inadequate nutrient intake or uptake that leads to altered body composition (e.g., reduced fat-free mass), diminished function, and worse clinical outcomes—also impacts prognosis (19). For instance, Rios-Olais et al. demonstrated that low muscle mass at diagnosis is associated with higher mortality in ALL, underscoring a correlation between nutritional status and survival (20). In hospitalized patients, malnutrition often stems not merely from inadequate intake but from disease-related pathophysiological mechanisms. Crucially, systemic inflammation and nutritional decline can form a self-perpetuating vicious cycle (21). Reflecting this complex interplay, ESPEN has classified malnutrition into three categories: disease-related malnutrition with inflammation, disease-related malnutrition without inflammation, and malnutrition without disease (19). Therefore, in this study, the inflammatory-nutritional indices CAR, CFA, and mGPS were selected to better capture the outcome of this interaction in T-ALL patients, rather than measuring inflammation or nutrition in isolation.

Our findings indicate that patients with a lower CAR, a lower CFA, or an mGPS of 0 at diagnosis were significantly more likely to achieve CR/CRi and MRD < 0.1% at EOI. These results suggest that a favorable inflammatory-nutritional profile at baseline may be associated with enhanced efficiency in disease eradication and bone marrow recovery. Notably, the prognostic impact of these indices extended to long-term survival outcomes. Patients in the high-risk groups (e.g., high-CAR, high-CFA or mGPS1-2) experienced significantly inferior OS and PFS. Multivariate analysis revealed both high CAR and MRD ≥ 0.1% at EOI as independent predictors of poor OS, underscoring the compounded adverse effects of inflammatory-nutritional profile and persistent residual disease on prognosis in T-ALL.

In line with our findings, the prognostic significance of inflammatory-nutritional indices is evident in diverse cancers. As summarized in Table 2, elevated CAR has been linked to poorer survival in lymphoma (22–24) and acute myeloid leukemia (AML) (13, 25). Similarly, the mGPS has been validated as a robust prognostic indicator in a range of solid tumors (26–42) and hematologic malignancies, including AML (43). Furthermore, a high CFA ratio has also been associated with inferior overall survival in AML (11, 43). The concordance of our results with these earlier studies collectively underscores the critical interplay between inflammatory-nutritional profile and survival across oncologic contexts.

**Table 2:** Summary of previous studies on CAR, CFA, and mGPS in solid and hematologic malignancies.

Index	Cancer type	Key findings	References
CAR (C-reactive protein to Albumin Ratio)	Head and neck cancer	Elevated CAR was associated with poor OS and DMFS.	(51–55)
	Lung cancer	Elevated CAR was correlated with poor survival.	(56, 57)
	Breast cancer	Elevated CAR was an independent predictor of poor DFS and CSS.	(58)
	Esophageal cancer	High pre-treatment CAR was an adverse prognostic factor for esophageal cancer.	(33, 59–61)
	Gastric cancer	High CAR was associated with shorter survival time.	(62–65)
	Hepatocellular carcinoma	High preoperative CAR was associated with poorer OS and DFS.	(66, 67)
	Biliary tract cancer	Elevated preoperative CAR predicted poor OS, independent of cutoff value, sample size, histology, or treatment.	(68)
	Pancreatic cancer	A higher CAR value was an independent and significant predictor of poor overall survival in patients undergoing pancreatic cancer resection.	(69–71)
	Colorectal cancer	High pre-treatment CAR was associated with poor OS and DFS in colorectal cancer.	(72–75)
	Urological cancer	High pre-treatment CAR was a predictor of poor survival in urinary cancers.	(76, 77)
	Gynecological cancer	High pre-treatment CAR was associated with poor OS, PFS, DFS and advanced-stage disease in gynecologic cancers.	(78–80)
	Lymphoma	High CAR at diagnosis was associated with poorer survival outcomes.	(22–24)
	Acute myeloid leukemia	High pre-induction chemotherapy CAR was independently associated with lower CR rates and poorer OS.	(13, 25)
CFA (CRP × fibrinogen/albumin ratio)	Acute myeloid leukemia	High CFA ratio was associated with poor OS.	(11, 43)
mGPS (modified glasgow prognostic score)	Head and neck cancer	High mGPS was associated with poorer PFS, OS and DFS.	(28)
	Lung cancer	High mGPS significantly impaired DCR, median PFS, and median OS.	(29, 30)
	Esophageal cancer	Elevated preoperative mGPS was significantly associated with worse OS.	(31–33)
	Gastric cancer	High mGPS level was significantly correlated with poor OS.	(34, 35)
	Pancreatic cancer	High mGPS level was significantly correlated with poor OS.	(36, 37)
	Colorectal cancer	mGPS is an effective prognostic indicator for OS and CSS in colorectal cancer.	(38)
	Urological cancer	High mGPS was associated with shorter survival time.	(39–42)
	Gynecological cancer	High mGPS was correlated with poor survival outcomes in gynecologic cancers.	(26, 27)
	Acute myeloid leukemia	High mGPS was associated with adverse outcomes in newly diagnosed AML.	(43)

OS, overall survival; DMFS, distant metastasis-free survival; DFS, disease-free survival; CSS, cancer-specific survival; PFS, progression-free survival; CR, complete remission; DCR, disease control rate; AML, acute myeloid leukemia.

A particularly insightful observation from our subgroup analysis relates to the role of HSCT. Although transplantation did not confer a significant survival benefit in the low-risk group, it markedly improved outcomes among high-risk patients. These findings suggest that the adverse prognosis associated with unfavorable inflammatory-nutritional profile may be partially mitigated by the potent graft-versus-leukaemia effect and myeloablative conditioning regimen inherent in HSCT. Therefore, early identification of high-risk patients using these readily accessible biomarkers could facilitate improved risk stratification and help select those most likely to benefit from intensive treatment strategies, including HSCT.

The biological plausibility of our findings is supported by the established literature. Albumin, which is primarily synthesized in the liver, not only serves as a marker of nutritional status but also has antiapoptotic signaling properties and participates in the transport and metabolism of chemotherapeutic agents in leukaemia (44). Xiao et al. reported that serum albumin levels were significantly associated with 60-day mortality in patients with *de novo* acute myeloid leukaemia (AML), with mortality risk increasing as albumin levels decreased (45). Additional studies have further corroborated that low serum albumin predicts higher cancer-related mortality (46). CRP is an acute-phase reactant protein synthesized by the liver in response to inflammatory cytokines such as IL-6. A large-scale prospective cohort study revealed that elevated baseline CRP levels were associated with an increased risk of subsequent cancer development (47). Elevated CRP levels serve as a direct marker of activation of the IL-6 signaling pathway, which has been implicated in promoting tumor cell survival, proliferation, and chemotherapy resistance (48). Luo et al. reported significantly elevated IL-6 activity in patients with ALL compared with that in healthy controls, and *in vitro* studies demonstrated that IL-6 promotes leukaemic cell proliferation (49). In addition to CRP, IL-6 also regulates fibrinogen production. Elevated plasma fibrinogen levels are significantly associated with increased mortality in patients with AML (50). Thus, the inflammatory-nutritional predictive indicators CAR, CFA, and mGPS which are composed of these common clinical biomarkers such as CRP, albumin, and fibrinogen represent low-cost and hold substantial value for prognostic assessment and treatment guidance in T-ALL patients.

Several limitations of our study should be acknowledged. First, its retrospective and single-centre design introduces potential selection bias and limits generalizability. Second, the sample size, although sufficient for initial observations, is modest, particularly for subgroup analyses. Third, we were unable to account for dynamic changes in CRP, CFA and mGPS during treatment, which might offer additional prognostic insights. Future prospective, multicentre studies with larger cohorts are needed to validate our findings and to explore whether targeted interventions to reduce inflammation or improve nutritional status can ameliorate the poor outcomes observed in high-risk patients.

## 5 Conclusion

The pretreatment CAR, CFA, and mGPS are simple, cost-effective, and robust prognostic tools for adults with T-ALL. They stratify patients into distinct risk groups with differential responses to induction chemotherapy and long-term survival outcomes. Integration of these indices into initial risk assessment may enhance prognostic accuracy and guide personalized treatment strategies, particularly in identifying candidates most likely to benefit from early and intensive interventions such as HSCT.

## Data Availability

The original contributions presented in the study are included in the article/supplementary material, further inquiries can be directed to the corresponding author.
